# Pathway analysis of clinical nurse educator’s intention to use virtual reality technology based on the UTAUT model

**DOI:** 10.3389/fpubh.2024.1437699

**Published:** 2024-11-15

**Authors:** Li Mengying, Fu Dongquan, Li Lin, Cao Yining, He Huijuan, Zhou Siyu, Yin Dan

**Affiliations:** ^1^School of Nursing, Hubei University of Chinese Medicine, Wuhan, Hubei Province, China; ^2^Hubei Shi Zhen Laboratory, Wuhan, Hubei Province, China

**Keywords:** virtual reality, behavioral intention, clinical nurse educator, UTAUT, pathway analysis

## Abstract

**Purpose:**

This study aims to investigate the willingness of clinical nurse educator to adopt virtual reality technology, while also examining the underlying mechanisms that influence this willingness through the lens of the Unified Theory of Acceptance and Use of Technology (UTAUT).

**Methods:**

A convenience sampling method was employed to select 225 clinical nurse educator, all of whom possess a professional qualification certificate as nurse practitioners, from a tertiary hospital in Wuhan City, Hubei Province. The study utilized an adapted UTAUT model theory-based design to develop several questionnaires: the performance expectancy questionnaire (11 items), the effort expectancy questionnaire (4 items), the social influence questionnaire (6 items), the facilitating conditions questionnaire (7 items), and the behavioral intention questionnaire (4 items). These instruments were designed to assess the clinical nurse educators’ willingness to adopt VR technology. Furthermore, a regression model was established to analyze the factors influencing this willingness, utilizing SPSS 26.0 for statistical analysis and validating the model through path analysis with AMOS 24.0, where a *p*-value of less than 0.05 was considered statistically significant.

**Results:**

The questionnaire demonstrated strong reliability and validity, yielding a total of 222 valid samples, comprising 209 females (94.14%) and 13 males (5.86%). Among the clinical nurse educators, 163 (73.42%) reported a willingness to use virtual reality technology, with scores of 4 or higher. Pearson correlation analysis revealed positive correlations between performance expectancy, effort expectancy, social influence, and facilitating conditions with behavioral intention (*p* < 0.05). Furthermore, regression analysis indicated that performance expectancy, effort expectancy, social influence, and facilitating conditions had a positive impact on behavioral intention (*p* < 0.05). The path model exhibited a good fit, and the results were consistent with the regression analysis, showing that the effects of performance expectancy, effort expectancy, and social influence on the behavioral intention to use virtual reality technology were 0.231, 0.150, 0.236, and 0.247, respectively.

**Conclusion:**

Clinical nurse educators exhibit a robust willingness to engage with VR technology. Moreover, improving factors such as performance expectancy, effort expectancy, social influence, and facilitating conditions can substantially enhance their readiness to adopt this technology.

## 1 Introduction

Nursing education serves as a cornerstone of the healthcare system, playing a vital role in public health and improving the quality of nursing services. However, traditional approaches to nursing education often prioritize the transmission of theoretical knowledge at the expense of developing practical skills. In clinical environments, students frequently face obstacles in obtaining sufficient hands-on experience due to resource constraints, as well as encountering occupational hazards such as exposure to infectious diseases and needlestick injuries (1). As a result, the investigation of innovative teaching methods and technologies has become an essential focus within the modern landscape of nursing education.

Virtual Reality (VR) technology, as an emerging innovation, has been extensively utilized in nursing education during the 21st century. It not only offers a safe and controlled learning environment but also simulates real clinical scenarios, enabling students to overcome the limitations inherent in traditional classrooms and clinical internships (2). This approach aids in enhancing their practical skills and their ability to navigate complex situations. Through VR technology, students can engage in various nursing procedures within a simulated context, which decreases the error rate in actual clinical settings while bolstering their self-confidence and professional competencies (3). Despite the promising prospects for VR’s application in nursing education, its widespread adoption faces several challenges. These challenges primarily include the reluctance of nursing educators to embrace new technologies, the high costs associated with VR systems, and the absence of comprehensive evaluation and feedback mechanisms that are essential for guiding the optimization and enhancement of this technology (4).

In response to these shortcomings, this study used the Unified Theory of Acceptance and Use of Technology (UTAUT) to examine the key factors affecting the intention to use virtual reality (VR) technology from the perspective of clinical nurse educators. This approach aims to increase acceptance of virtual reality among clinical nurse educators and promote its use in nursing education (5). The UTAUT model offers a comprehensive theoretical framework that elucidates and predicts individual technology acceptance provides behavior through four core constructs: performance expectancy, effort expectancy, social influence, and facilitating conditions. Performance expectancy refers to the anticipated benefits derived from the technology, while effort expectancy emphasizes the ease of use associated with the technology, Social influence relates to the perceptions of others regarding technology adoption, and facilitating conditions encompass the organizational and technological infrastructure that supports technology usage. Together, these constructs significantly impact an individual’s behavioral intention, subsequently influencing their actual intention to utilize the technology (6).

This study is based on the UTAUT model and aims to explore the potential mechanisms that influence clinical nurse educators: willingness to adopt VR technology. It aims to promote the comprehensive integration of VR technology into nursing education, thereby improving the quality and efficiency of the entire nursing education field. We hope to integrate more innovative elements into nursing education through research, promote the modernization of nursing education, and ultimately improve the quality of nursing services.

## 2 Methods

### 2.1 Participants

To reduce sampling costs and ensure the acquisition of sufficient and representative samples, the convenience sampling method was employed to select the clinical nurse educator who had obtained the professional qualification certificate of nurse practitioner from a tertiary hospital in Wuhan City, Hubei Province as the research object. Inclusion criteria for this study comprised in-hospital clinical nurse educators who possess a nurse professional qualification certificate and who provided informed consent to voluntarily participate. Exclusion criteria included individuals who have not engaged in any teaching activities within the past year and those who withdrew from the study midway. Breckler’s study indicates that a sample size exceeding 200 can be utilized to construct a more stable equation model (7). The survey was distributed via the Questionstar platform, with a total of 225 questionnaires being disseminated.

### 2.2 Instruments

#### 2.2.1 Demographic information

A total of five entries were included in the study, comprising information on gender, age, levels of education, years of work experience, and whether or not the participants had been exposed to VR technology.

#### 2.2.2 Clinical nurse educators’ willingness to use virtual reality technology questionnaire

This questionnaire was adapted from the questionnaire designed by Dandan Lu et al. (8),based on the Unified Theory of Acceptance and Use of Technology (UTAUT), namely “Clinical Nurse Educators’ Willingness to Use Virtual Simulation Technology.” The adapted questionnaire consists of five parts, each employing the forward scoring method and utilizing a 5-point Likert scale. The scoring ranges from 5 to 1, reflecting responses from ‘completely agree’ to ‘completely disagree.’ The overall Cronbach’s *α* coefficient is 0.964, which exceeds the acceptable threshold of 0.700, indicating high internal consistency. Furthermore, the KMO value is 0.960, also above the threshold of 0.700, and the results of Bartlett’s sphericity test yield a significance level of *p* < 0.01, confirming the questionnaire’s robust reliability and validity. Selected variables and measurement items are detailed in Table 1, while a comprehensive list of all measurement items is provided in Appendix 1.

1. Performance expectancy, consisting of 11 items, delineate the advantages that clinical nurse educators can derive from the application of VR technology in their practice. A higher score indicates greater perceived utility in their work. In this study, Cronbach’s ⍺ = 0.954 > 0.700, KMO value = 0.966 > 0.700, Bartlett sphericity test *p* < 0.01.
2. Effort expectancy, consisting of 4 items, reflects the ease of utilizing virtual reality technology, including the challenges associated with locating teaching materials and performing technical operations. A higher score indicates greater ease of use, while a lower score suggests increased difficulty. In this study, Cronbach’s ⍺ = 0.894 > 0.700, KMO value = 0.846 > 0.700, Bartlett’s sphericity test *p* < 0.01.
3. Social influence, consisting of 6 items, refers to the level of support for the use of virtual reality technology by individuals or groups that clinical nurse educators deem significant. This support may manifest as mutual encouragement among colleagues. A higher score indicates a greater degree of support. In this study, Cronbach’s ⍺ = 0.932 > 0.700, KMO value = 0.929 > 0.700, Bartlett sphericity test *p* < 0.01.
4. Facilitating conditions, consisting of 7 items, refers to the level of support perceived by clinical nurse educators regarding hardware, funding, technical training, and other resources necessary to promote the use of VR technology. Higher scores reflect greater levels of support. In this study, Cronbach’s ⍺ = 0.933 > 0.700, KMO value = 0.941 > 0.700, Bartlett sphericity test *p* < 0.01.
5. Behavioral intention, consisting of 4 items, refers to the behavioral tendency of clinical nurse educators to adopt virtual reality technology. This tendency is evidenced by their willingness to utilize virtual reality technology, their intention to increase its usage, and their readiness to recommend it to colleagues. A higher score indicates a greater propensity to use virtual reality technology. The willingness to use is categorized into two groups based on a cut-off value of 4 points: a score of ≥4 points signifies a high willingness to use and is classified as the high group, while a score of <4 points indicates a generally low willingness to use. In this study, Cronbach’s ⍺ = 0.883 > 0.700, KMO value = 0.831 > 0.700, Bartlett sphericity test *p* < 0.01.

**Table 1:** Variables and partial sample measurement questions.

Variables	Meaning	Measurement questions
Performance expectancy	The benefits of using VR technology for your own work	1. It makes my teaching more interesting
		2. I am satisfied with the use of virtual reality in nursing education
		3. It gives me a sense of fulfillment at work
Effort expectancy	Ease of using VR technology	1. My search for virtual reality based teaching materials is not very difficult
		2. It is not difficult for me to design the content of teaching virtual reality technology that fits the speciality of my profession
		3. I learnt that teaching operations using virtual reality technology is not difficult
Social influence	Degree of support for one’s use of virtual reality technology from individuals or groups that one considers important to oneself	1. My colleague suggested that I use virtual reality technology in my teaching
		2. Students expect me to use virtual reality in teaching and learning
		3. Schools, students and others rate the use of virtual reality technology in nursing education highly
Facilitating conditions	Level of support for hardware, finance, technical training, etc. to promote the use of VR technology	1. I have the hardware equipment and facilities to use virtual reality technology in teaching and learning, e.g., computers, networks, venues, virtual glasses, etc.
		2. I was able to get funding and other support for teaching virtual reality technology
		3. My college or department has a better team for teaching and researching virtual reality technology
Behavioral intention	Behavioral tendencies to use VR technology	1.I prefer the use of virtual reality in nursing education
		2.I would like to use virtual reality in nursing education
		3.I would recommend the use of virtual reality technology in nursing education to colleagues in my neighborhood

## 3 Data collection

The questionnaire designed for this study includes demographic information and five measurement variables based on the UTAUT theoretical model: performance expectancy, effort expectancy, social influence, facilitating conditions and behavioral intention. The production of the questionnaire is facilitated by the Questionstar platform, and the questionnaires are distributed online. Each respondent is allowed to submit only one questionnaire.

## 4 Statistical analyses

To ensure the reliability of the data and the accuracy of the results, we eliminated incompletely filled questionnaires and those with unchanged responses prior to data analysis. The coded data were analyzed using SPSS 26.0 and AMOS 24.0 statistical software. Initially, we conducted basic reliability and validity tests on the questionnaire, employing Cronbach’s *α* coefficient to assess reliability. Bartlett’s test of sphericity and the KMO value were used to evaluate validity. Subsequently, demographic and sociological data, along with general information, were analyzed using descriptive statistics. Measurement data were reported as mean ± standard deviation, while categorical data were expressed as frequency and percentage. To assess the factors influencing the willingness to utilize virtual reality technology, we first categorized the willingness scores into high and low groups based on a 4-point cutoff. Following this, we performed a multi-factor logistic regression analysis to examine the relationship between demographic and sociological factors and the willingness to use virtual reality technology. To investigate the relationship between the variables based on the Unified Theory of Acceptance and Use of Technology (UTAUT) and willingness to use, we tested the data for normality to ascertain the distribution of performance expectancy, effort expectancy, social influence, facilitating conditions and behavioral intention. If the data exhibited a normal distribution or was approximately normal, we employed Pearson correlation analysis to examine the correlations among performance expectancy, effort expectancy, social influence, facilitating conditions and behavioral intention. Conversely, if the variables did not meet the normality condition, we opted for Spearman correlation analysis to further investigate the impact of UTAUT-based variables on usage intention. We also performed a multicollinearity test (Variance Inflation Factor, VIF) on the variables. If no multicollinearity was detected among performance expectancy, effort expectancy, social influence, facilitating conditions and behavioral intention, we included the variables related to intention to use in the stepwise regression analysis. In cases where multicollinearity was present, we employed Ridge regression analysis. Finally, to further identify the variables affecting usage intention, we constructed a path model following confirmatory factor analysis using AMOS 24.0 statistical software to validate the effectiveness of the regression model. *p* < 0.05 was considered statistically significant.

## 5 Results

A total of 225 electronic questionnaires were distributed through questionnaire star, excluding 3 questionnaires whose response time was too short, 222 valid questionnaires were recovered, with an effective recovery rate of 98.67%. This meets the requirements for basic data analysis and the analysis was conducted by using SPSS 26.0 and AMOS 24.0. The results are as follows:

### 5.1 General information about clinical nurse educators

In terms of gender, there were 209 (94.14%) female teachers and 13 (5.86%) male teachers. More than half of the clinical nurse educators were under 40 years of age, 122 (55%). There were 54 (24.32%) senior nurse teachers with 5 and less years of experience, 99 (44.60%) with 6 to 15 years of experience, 49 (22.07%) with 16 to 30 years of nursing experience and 20 (9.01%) with 31 and more years of nursing experience. There were 138 (62.16%) who had been exposed to VRT and 84 (37.84%) who had not been exposed to VRT, and detailed data are shown in Table 2.

**Table 2:** General information on clinical nurse educators (*N* = 222).

Variables		Number	%
Gender	Female	209	94.14
	Male	13	5.86
Age (Years)	≤30	51	22.97
	31–40	71	31.98
	41–50	71	31.98
	≥51	29	13.06
Education level	Bachelor’s degree or other	133	59.91
	Master’s degree or PhD	89	40.09
Years of working experience (Years)	≤5	54	24.32
	6 ~ 15	99	44.60
	16 ~ 30	49	22.07
	≥31	20	9.01
Whether exposed to virtual reality technology	Yes	138	62.16
	No	84	37.84

### 5.2 Analysis of the current status of clinical nurse educators’ willingness to use virtual reality technology

Following the normality test, the data performance expectancy, effort expectancy, social influence, facilitating conditions exhibited a kurtosis close to 3, a skewness close to 0, and a data distribution that was approximately normal. The means and standard deviations of the scores for each variable were as follows: the highest scoring variable was behavioral intention, with a mean and standard of 3.87 ± 0.85; effort expectancy score was the lowest with a mean and standard of 3.54 ± 0.89. A total of 163 (73.42%) scored a behavioral intention score of ≥4, indicating that 73.42% of clinical nurse educators in this sample exhibited a higher willingness to use VR technology. This is illustrated in Table 3.

**Table 3:** Current status of clinical nurse educators’ willingness to use VR technology.

Variables	Number of entries	Score(^−^x ± s)
Behavioral intention	4	3.87 ± 0.85
Facilitating conditions	7	3.57 ± 0.88
Social influence	6	3.79 ± 0.92
Effort expectancy	4	3.54 ± 0.89
Performance expectancy	11	3.76 ± 0.83

### 5.3 Multifactor logistic regression analysis of demographic and sociological characteristics on willingness to use VR technology

The following variables were used as independent variables: gender, age, education levels, years of work experience, and whether or not they had been exposed to virtual reality technology. Multifactorial logistic regression analysis was performed with behavioral intention as the dependent variable, with scores greater than or equal to 4 being classified as a high group and less than 4 as a low group. The results demonstrated that there was no statistically significant difference in the willingness to use virtual reality technology among clinical nurse educators of different genders, ages, levels of education, and years of experience (*p* > 0.05). Furthermore, the impact of prior exposure to virtual reality technology on the willingness to use VR technology was not statistically significant (*p* > 0.05), as shown in Table 4.

**Table 4:** Results of multifactor logistic regression analysis of sociological characteristics of the population and behavioral intention.

Variables	B	SE	Wald	*P*	Exp(B)	EXP(B) 95% CI	
						lower limit	limit
Gender (as reference: male)							
Female	0.318	0.653	0.238	0.626	1.375	0.382	4.942
Age (as reference: ≥51)							
≤30	1.183	1.012	1.368	0.242	3.265	0.450	23.715
31–40	0.711	0.772	0.848	0.357	2.036	0.448	9.249
41–50	0.895	0.871	1.056	0.304	2.448	0.444	13.504
Education level (as reference: Master’s degree or PhD)							
Bachelor’s degree or other	−0.160	0.376	0.181	0.670	0.852	0.408	1.780
Years of working experience (as reference: ≥31)							
6–15	−0.533	0.961	0.307	0.580	0.587	0.089	3.864
16–30	−0.688	0.834	0.681	0.409	0.503	0.098	2.575
≤5	−1.147	1.144	1.006	0.316	0.317	0.034	2.988
Whether exposed to virtual reality technology (as reference: Yes)							
No	−0.253	0.316	0.643	0.423	0.776	0.418	1.442

### 5.4 Analysis of the impact of variables on behavioral intention based on the UTAUT model

#### 5.4.1 Person-related analyses

**Figure 1:**
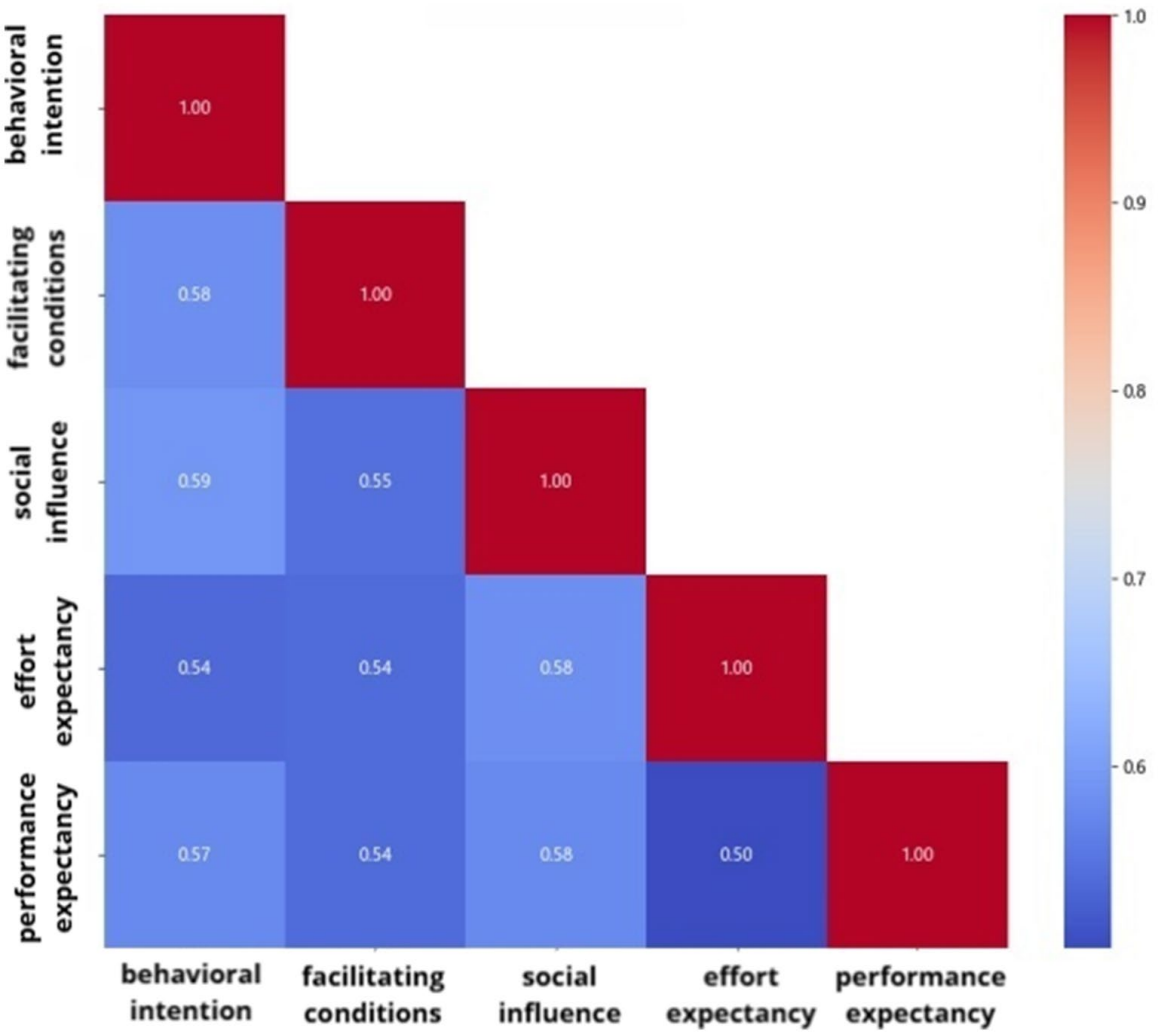
Correlation analysis results of each variable in the study on the willingness of clinical nurse educator to use virtual reality technology.

Following the normality test, the data performance expectancy, effort, expectancy, social influence, facilitating conditions and behavioral intention were found to be approximately normally distributed. The results of correlation analyses demonstrated that performance expectancy, effort expectancy, social influence, facilitating conditions and behavioral intention were positively correlated, *p* < 0.05, with R-values of 0.58, 0.59, 0.54, and 0.57, respectively, as shown in Figure 1.

#### 5.4.2 Stepwise regression analysis of variables and behavioral intention in the UTAUT model

After multicollinearity testing, the VIF values are all less than 3. There is no multicollinearity problem between variables, and the data is good. In order to clarify the relationship between performance expectancy, effort expectancy, social influence, facilitating conditions and behavioral intention in the UTAUT model, multiple stepwise regression analysis was employed. Performance expectancy, effort expectancy, social influence, and facilitating conditions were used as independent variables, and behavioral intention was used as the dependent variable in the stepwise regression analysis. After four iterations, the best model 4 was obtained, and four factors entered the regression equation, as detailed in Table 4. The final regression equation was established as follows: behavioral intention = 0.783 + 0.22*social influence +0.239*facilitating conditions+0.236*performance expectancy+0.144*effort expectancy. See Tables 5, 6.

**Table 5:** Results of stepwise regression model analysis.

Model	R	R2	Adjusted R2	SE	Durbin Watson	F	*p*
1	0.591^b^	0.350	0.347	0.688	1.990	118.301	0.000^b^
2	0.667^c^	0.445	0.440	0.638		87.749	0.000^c^
3	0.695^d^	0.484	0.477	0.616		68.084	0.000^d^
4	0.705^e^	0.497	0.487	0.610		53.549	0.000^e^

b Predictor variable: (constant), Social influence.

c Predictor variables: (Constant), Social influence, Facilitating conditions.

d Predictor: (Constant), Social Influence, Facilitating conditions, Performance expectancy.

e Predictor variables: (Constant), Social Influence, Facilitating conditions, Performance expectancy, Effort expectancy.

**Table 6:** Regression coefficient test.

Variables	Unstandardized coefficient		Standardized coefficient beta	*t*	Sig.	95.0% CI		collinearity statistics	
	*B*	standard error				lower limit	limit	Tolerance	VIF
(Constant)	0.783	0.216		3.623	<0.001	0.357	1.209	0.526	1.900
Social influence	0.220	0.062	0.236	3.560	<0.001	0.098	0.341	0.584	1.712
Facilitating conditions	0.239	0.061	0.247	3.919	<0.001	0.119	0.359	0.581	1.722
Performance expectancy	0.236	0.065	0.231	3.651	<0.001	0.109	0.364	0.575	1.738
Effort expectancy	0.144	0.061	0.150	2.370	0.019	0.024	0.264	0.526	1.900

#### 5.4.3 Path analysis of variables and behavioral intention of UTAUT model

##### 5.4.3.1 Confirmatory factor analysis

**Figure 2:**
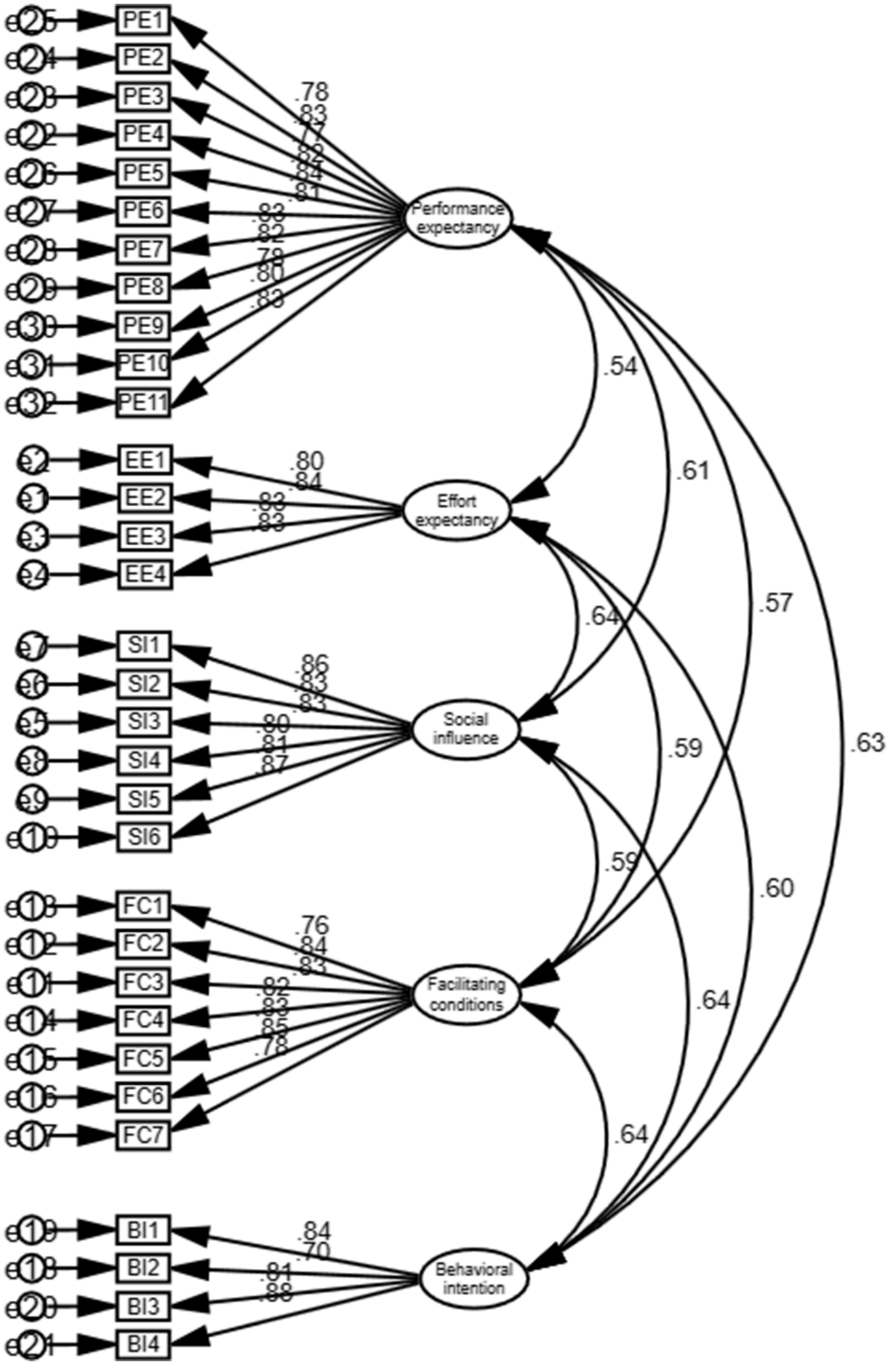
Confirmatory factor analysis measurement model diagram.

As illustrated in Figure 2, the AMOS software was utilized to create the confirmatory factor analysis measurement model diagram. In this model, the CMIN/DF = 1.135 and the RMSEA = 0.025, which is below the threshold of 0.08, indicating a well-constructed model. At a significance level of *p* < 0.001, the factor loadings of all observed variables are significant, with standardized factor loading coefficients exceeding 0.7 and average variance extracted (AVE) values greater than 0.5. Consequently, this measurement model demonstrates strong convergent validity, as shown in Table 7. The square roots of the AVE for performance expectancy, effort expectancy, social influence, facilitating conditions, and behavioral intention variables are 0.812, 0.826, 0.837, 0.817, and 0.819, respectively. All of these values surpass the Pearson correlation coefficients among the variables, thereby confirming that this measurement model possesses high discriminant validity, as presented in Table 7.

**Table 7:** Confirmatory factor analysis results.

Path name	Factor loading coefficient	S.E	*p*	CR	AVE
Performance expectancy→PE1	0.776			0.955	0.659
Performance expectancy→PE2	0.831	0.077	***		
Performance expectancy→PE3	0.770	0.071	***		
Performance expectancy→PE4	0.824	0.074	***		
Performance expectancy→PE5	0.841	0.078	***		
Performance expectancy→PE6	0.806	0.078	***		
Performance expectancy→PE7	0.828	0.079	***		
Performance expectancy→PE8	0.823	0.083	***		
Performance expectancy→PE9	0.781	0.078	***		
Performance expectancy→PE10	0.800	0.083	***		
Performance expectancy→PE11	0.834	0.082	***		
Effort expectancy→EE1	0.802			0.895	0.682
Effort expectancy→EE2	0.840	0.070	***		
Effort expectancy→EE3	0.830	0.065	***		
Effort expectancy→EE4	0.835	0.072	***		
Social influence→SI1	0.858			0.933	0.700
Social influence→SI2	0.830	0.060	***		
Social influence→SI3	0.830	0.060	***		
Social influence→SI4	0.798	0.058	***		
Social influence→SI5	0.814	0.062	***		
Social influence→SI6	0.874	0.064	***		
Facilitating conditions→FC1	0.760			0.933	0.668
Facilitating conditions→FC2	0.843	0.082	***		
Facilitating conditions→FC3	0.831	0.087	***		
Facilitating conditions→FC4	0.822	0.087	***		
Facilitating conditions→FC5	0.825	0.085	***		
Facilitating conditions→FC6	0.845	0.086	***		
Facilitating conditions→FC7	0.781	0.078	***		
Behavioral intention→BI1	0.843			0.889	0.671
Behavioral intention→BI2	0.705	0.066	***		
Behavioral intention→BI3	0.810	0.073	***		
Behavioral intention→BI4	0.877	0.073	***		

***represents *p* < 0.001.

##### 5.4.3.2 Path analysis

**Figure 3:**
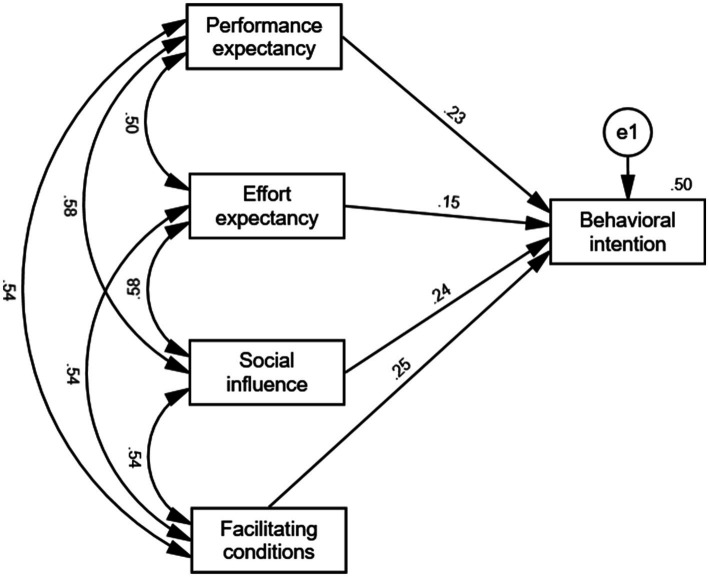
Path structure diagram.

To further validate the regression model, we established a path depicting the factors influencing nursing clinical teachers’ willingness to use virtual reality technology, as illustrated in Figure 3. The model demonstrates a good fit, with RMR = 0.00 < 0.08, CFI = 1.00 > 0.90, and IFI = 1.00 > 0.90. The effect sizes of performance expectancy, effort expectancy, social influence, and facilitating conditions on the behavioral intention are 0.231, 0.150, 0.236, and 0.247, respectively. Notably, facilitating conditions exert the largest impact, followed by social influence. The standardized path coefficients of the model are consistent at 0.231, 0.150, 0.236, and 0.247, with all *p* values being less than 0.05, as detailed in Table 8.

**Table 8:** Table of path coefficients.

Path name	Estimate	S.E.	C.R.	*p*
Performance expectancy→behavioral intention	0.231	0.064	3.684	<0.001
Effort expectancy→behavioral intention	0.150	0.060	2.392	0.017
Social influence→behavioral intention	0.236	0.061	3.593	<0.001
Facilitating conditions→behavioral intention	0.247	0.060	3.955	<0.001

## 6 Discussion

### 6.1 There are no discernible differences in the willingness of clinical nurse educators with varying demographic and sociological characteristics to utilize virtual reality technology

The results of this study indicate that there is no statistically significant difference in the willingness to use VR technology among clinical nurse educators based on gender, age, education level, or years of experience. In the study conducted by Hyun et al., it was found that gender significantly influences consumers’ enjoyment and willingness to replay VR games, with men exhibiting a greater propensity to enjoy and replay these games compared to their female counterparts (9). This discrepancy may be attributed to the specific application context of virtual reality technology. The virtual reality nursing context of the present study is not gender-biased, aligning with the findings that gender does not impact the willingness of nursing clinical teachers to utilize virtual reality. Nevertheless, future research should thoroughly investigate the factors influencing the willingness to adopt virtual reality. Given the research context, it is recommended that additional demographic and sociological factors, as well as sample characteristics, be incorporated to better understand the individual traits that may affect the willingness to use VR technology. This study did not find evidence that prior exposure to virtual reality technology affects individuals’ willingness to use it. This finding is consistent with the conclusion drawn by Cao Xiao-yue in her research on the willingness of virtual reality game players, which similarly indicates that prior exposure does not significantly influence their willingness to engage with the technology (10). However, this contrasts with the findings of Lu Dandan et al., who investigated the impact of virtual simulation technology in medical education and found that it does affect medical teachers’ willingness to adopt such technology (8). The differing outcomes regarding the influence of exposure to virtual reality technology on willingness to use it may be attributed to the specific contexts in which the technology is utilized. Efiloğlu Kurt et al. (11) demonstrated that students’ willingness to use virtual reality technology varies between Turkish and British learning environments, with distinct influencing factors in each context. Furthermore, Riva et al. (12) highlighted that individuals experience different emotions or values in varied environments. It is possible that these diverse experiences contribute to the differing results regarding the impact of exposure on the intention to use VR. Therefore, future research on the intention to use virtual reality technology should consider the specific contexts of its application to better understand how varying usage scenarios affect willingness to use before and after exposure to virtual reality, ultimately enhancing the research model on the intention to use this technology.

### 6.2 Performance expectancy and effort expectancy influence clinical nurse educators’ willingness to use VR technology

In this study, performance expectancy positively influence the intention to use VR technology. This finding aligns with the results of previous research and is consistent with the UTAUT model, which suggests that clinical nurse educators are more inclined to adopt virtual reality technology if they perceive it as beneficial for their work (13). The implementation of VR technology can effectively mitigate risks, such as needle sticks and infections, that clinical nurse educators and students encounter in actual clinical settings. Furthermore, enhancing the nursing operational skills of both teachers and students through virtual training represents a crucial advancement in nursing clinical safety education. Additionally, compared to traditional teaching methods, VR technology training not only enhances students’ learning performance more efficiently but also makes the learning process more engaging. Therefore, VR technology, characterized by its high efficiency and safety, is increasingly appealing to both teachers and students for educational purposes. However, the full potential of VR technology has yet to be realized, particularly in the context of clinical teaching hospitals, where its application remains limited. To enhance the willingness to adopt VR technology and to promote its integration into clinical education, a teaching plan can be developed that facilitates collaborative observation and engagement between teachers and students. This approach would allow them to experience the enjoyment and effectiveness that VR technology offers, thereby enhancing their perception of its potential benefits (14). Additionally, developers of VR equipment could increase awareness by implementing promotional strategies and offering free trial programs, providing more teachers and students with opportunities to explore and understand this technology. By implementing these measures, the acceptance and willingness of both teachers and students to embrace VR technology can be significantly improved, ultimately fostering its broader application in clinical education.

Effort expectancy positively influences the behavioral intention of using VR technology. This research finding aligns with the model proposed by Cao Ruiyang et al., which examines factors affecting learners’ acceptance of virtual experiment platforms based on the UTAUT model (15). Effort expectancy serves as an indicator of the ease of using technology; in this study, a higher effort expectancy score correlates with greater ease of operation and use of VR technology. The simplest form of human-computer interaction can be achieved through a computer and mouse. However, for highly immersive virtual reality experiences, specialized display helmets are also utilized. As VR technology continues to evolve, the interaction and operation will become increasingly user-friendly, and virtual reality teaching software will become more accessible, particularly with the growth of computer expertise. Consequently, educators are more likely to adopt this technology. Nonetheless, certain technical barriers remain, necessitating deeper collaboration and strengthened technical exchanges and resource sharing between teaching hospitals and universities. Furthermore, partnerships between educational institutions and development units are essential; these initiatives are critical for advancing the application of virtual reality technology in nursing education.

In related research on the acceptance of VR technology, performance expectancy is typically the decisive factor influencing willingness to accept such technology (16). In this study, effort expectancy (*β* = 0.150) demonstrates the smallest impact on the behavioral intention, whereas performance expectancy has a greater impact (*β* = 0.231); however, it is not the sole decisive factor in the intention to use VR technology. Despite the significant advantages of VR technology in educational and teaching applications, challenges persist regarding teacher adaptation and technological integration during the implementation process (17). These challenges may contribute to the relatively low influence of performance expectancy on the behavioral intention. Conversely, this outcome could also be linked to individuals’ exposure to various types of VR systems, as differing levels of immersion and ease of operation can lead to varied experiences, thereby affecting perceptions of the enjoyment and effectiveness of virtual reality technology. Future research should incorporate additional factors relevant to the research context in order to investigate the elements that influence performance expectancy, effort expectancy, social influence, and facilitating conditions. This approach will contribute to a more comprehensive understanding of how clinical nurse educators utilize VR technology. In light of the adaptation and technical challenges associated with integrating VR technology into nursing education, it is essential for nursing clinical teachers to participate in the development process of nursing VR technology. This involvement will enable teachers to gradually adjust to new teaching methodologies and become proficient in system operation during the development phase. Furthermore, an operational training system designed in this manner can better align with the clinical application scenarios in nursing, thereby enhancing the acceptance and practicality of VR technology within nursing education.

### 6.3 Social influence and facilitating conditions influence clinical nurse educators’ willingness to use VR technology

Social influence positively affects the behavioral intention of using virtual reality technology. This finding aligns with the research results of other scholars (18) and is consistent with the UTAUT model. It indicates that the greater the support an individual receives from significant individuals or groups regarding the use of virtual reality technology, the stronger their intention to adopt it (19). Such support may stem from recommendations and encouragement from colleagues, as well as from policies enacted by the country, educational institutions, and teaching hospitals. The influence of facilitating conditions on the intention to use is both significant and positive. This result mirrors the findings of Zhang Yingjie et al. (20) and Gunasinghe et al. (21), suggesting that the more external resources—such as hardware, funding, and technical support—an individual receives, the greater their willingness to utilize virtual reality technology. In this study, facilitating conditions exhibited the most substantial impact on the willingness to use virtual reality technology (*β* = 0.247), followed closely by social influence. Whether through volitional support or material encouragement, the degree of support remains a critical factor in the willingness to adopt VR technology. In the context of increasing informatization and intelligent education, VR technology has garnered significant attention not only in universities but also in large tertiary hospitals, where the application of virtual surgery and 3D anatomy is thriving. The advancement of medical VR technology can facilitate the integration of VR into nursing education to a considerable extent. Concurrently, educational authorities at all levels have introduced policies that provide financial support and establish related initiatives to promote the application of VR technology in educational settings. Consequently, as societal developments continue, facilitating conditions and social influence are expected to rise, thereby enhancing the willingness of clinical nurse educators to adopt VR technology. It is essential for clinical nurse educators to strengthen collaboration in experience sharing, teaching, and other areas to cultivate a positive community atmosphere. Furthermore, organizations that implement incentive mechanisms and offer hardware, financial, and other forms of support represent effective strategies to bolster educators’ willingness to utilize virtual reality technology, thereby further advancing its application in clinical teaching.

## 7 Conclusion

This study found that clinical nurse educators exhibit a high willingness to utilize virtual reality technology, with 73.42% of participants scoring 4 points or above on their willingness assessment. The research draws upon the classic variables of the UTAUT model to design its variables, exploring the factors that influence clinical nurse educators’ willingness to adopt VR technology and establishing a path model. Consequently, enhancing performance expectancy, effort expectancy, social influence, and facilitating conditions for clinical nurse educators can significantly increase their willingness to use virtual reality technology. This, in turn, may assist teaching hospitals in fostering greater acceptance of VR technology among clinical nurse educators, thereby providing a foundation for the further promotion of this innovative technology. Additionally, the study offers reference opinions on the application of VR technology in clinical nurse education.

## 8 Limitations and future prospects

The respondents of this study were all from a tertiary teaching hospital, which may limit the generalizability of the study results. Future research should aim to expand the sample size, validate findings across multiple regions, and consider incorporating interviews or other qualitative research methods to gain a deeper understanding of the mechanisms underlying each variable. Additionally, this study employed a convenience sampling method, which may introduce certain biases. Future investigations should utilize more rigorous sampling techniques, such as random or stratified sampling, to obtain more representative data from various hospitals across different regions. This study utilized a cross-sectional design, which precludes the determination of causal relationships or trends. To address this limitation, future research could adopt a longitudinal design to track changes within the same group of participants, thereby enhancing our understanding of the behavioral development of clinical nurse educators using VR technology. The demographic information collected in this study is relatively limited; future research should include a broader range of demographic data, such as age, gender, educational background, and technology proficiency, to explore how these factors influence clinical nurse educators’ acceptance of virtual reality technology. Furthermore, this study is grounded in a specific technology acceptance model. Future research could integrate additional relevant theoretical frameworks of technology acceptance to develop a more comprehensive model that elucidates clinical nurse educator’s willingness to utilize VR technology.

## Data Availability

The original contributions presented in the study are included in the article/Supplementary material, further inquiries can be directed to the corresponding author.
