# Supplementary material for: Pathway analysis of clinical nurse educator’s intention to use virtual reality technology based on the UTAUT model
Source: Front Public Health. 2024 Nov 15;12:1437699. doi: 10.3389/fpubh.2024.1437699 (PMC11604721; doi:10.3389/fpubh.2024.1437699)
Supplement: Supplementary file 1 [file Data_Sheet_1.docx]

Attachment 1:

Dear teacher:

Greetings! This survey hopes to understand your willingness to use virtual reality technology. This questionnaire is anonymous. There is no right or wrong answer. The survey results are for research purposes only and will not have any adverse impact on you. If you To agree to participate in this survey, please fill it out independently based on your true situation and thoughts. Your answers are very important to our research, thank you for your assistance and support!.

Part 1: Demographic and Sociological Information

1. Gender

○ Female ○ Male

1. Age

○ 30 years old and below ○ 31~40 years old ○ 41~50 years old ○ 51 years old and above

1. Educational level

○ Bachelor’s degree or other ○ Master’s or PhD Graduate students

1. Working experience

○ 5 years and less ○ 6 to 15 years ○ 16 to 30 years ○ 31 years and above

1. Have you been exposed to virtual reality technology

○ Yes ○ No.

Part 2 UTAUT Questionnaire

| **Variables** | **Measurement questions** |
| --- | --- |
| **Performance expectancy** | 1.The application of VR technology can compensate for scenarios that are not feasible in traditional teaching, particularly those involving high-risk, high-cost, and long-duration content or processes. |
|  | 2.Able to break the limitations of teaching space and time and innovate teaching models. |
|  | 3.Make the teaching process more vivid and vivid. |
|  | 4.Can improve teaching effectiveness and easily achieve teaching goals. |
|  | 5.Conveniently and comprehensively record and evaluate students’ learning process. |
|  | 6.In short, using virtual reality technology is helpful for me to teach. |
|  | 7.It gives me a sense of fulfilment at work |
|  | 8.It can lead to more communication and interaction between me and students. |
|  | 9.It makes my teaching more interesting |
|  | 10.The virtual world created through virtual reality technology is interesting. |
|  | 11.I am satisfied with the use of virtual reality in nursing education |
| **Effort expectancy** | 1.My search for virtual reality based teaching materials is not very difficult |
|  | 2.It is not difficult for me to design the content of teaching virtual reality technology that fits the speciality of my profession |
|  | 3.I learnt that teaching operations using virtual reality technology is not difficult |
|  | 4.All in all, it's not hard for me to teach using virtual reality technology |
| **Social influence** | 1.The development trend of nursing education in the context of information technology in education and the construction of first-class courses require me to use VR technology in teaching. |
|  | 2.National policy supports my use of virtual reality technology in teaching. |
|  | 3.School and college policies support my use of virtual reality technology in teaching. |
|  | 4.My colleague suggested that I use virtual reality technology in teaching. |
|  | 5.Students expect me to use virtual reality technology in teaching. |
|  | 6.Schools, students, etc. have high evaluations of the application of virtual reality technology in nursing education. |
| **Facilitating conditions** | 1.I have the hardware equipment and facilities to use virtual reality technology in teaching, such as computers, networks, venues, virtual glasses, etc. |
|  | 2.I was able to obtain funding and other support for virtual reality technology teaching. |
|  | 3.My college or department has a good virtual reality technology teaching and research team. |
|  | 4.My college or department has long-term cooperation with technical production companies. |
|  | 5.I had the opportunity to participate in exchanges and learning about the application of virtual reality technology in teaching. |
|  | 6.If I encounter problems using virtual reality technology in teaching, I can get technical guidance or find solutions in time. |
|  | 7.I have the knowledge and skills to use virtual reality technology in teaching. |
| **Behavioral intention** | 1.I prefer using virtual reality technology in nursing education. |
|  | 2.I am willing to use virtual reality technology in nursing education. |
|  | 3I will increase the use of virtual reality technology in nursing education in the future. |
|  | 4.I am willing to recommend the application of virtual reality technology in nursing education to my colleagues around me. |
